# Association Between Vaccination Status and Mortality Among Intubated Patients With COVID-19–Related Acute Respiratory Distress Syndrome

**DOI:** 10.1001/jamanetworkopen.2022.35219

**Published:** 2022-10-07

**Authors:** Eirini Grapsa, Georgios Adamos, Ioannis Andrianopoulos, Vasiliki Tsolaki, Vassilis G. Giannakoulis, Nikitas Karavidas, Vassiliki Giannopoulou, Katerina Sarri, Eleftheria Mizi, Evdokia Gavrielatou, Georgios Papathanakos, Konstantinos D. Mantzarlis, Zafeiria Mastora, Eleni Magira, Vasilios Koulouras, Anastasia Kotanidou, Ilias I. Siempos

**Affiliations:** 1 First Department of Critical Care Medicine and Pulmonary Services, Evangelismos Hospital, National and Kapodistrian University of Athens Medical School, Athens, Greece; 2 Department of Intensive Care Unit, University Hospital of Ioannina, Ioannina, Greece; 3 Critical Care Department, University Hospital of Larissa, Faculty of Medicine, University of Thessaly, Larissa, Greece; 4 Department of Medicine, Division of Pulmonary and Critical Care Medicine, Weill Cornell Medicine, New York, New York

## Abstract

**Importance:**

Although vaccination substantially reduces the risk of severe COVID-19, it is yet unknown whether vaccinated patients who develop COVID-19 and require invasive mechanical ventilation have lower mortality than controls.

**Objective:**

To examine the association between COVID-19 vaccination status and mortality among critically ill patients who require invasive mechanical ventilation owing to acute respiratory distress syndrome (ARDS) related to COVID-19.

**Design, Setting, and Participants:**

This multicenter cohort study was performed between June 7, 2021, and February 1, 2022, among 265 consecutive adult patients with COVID-19 in academic intensive care units who underwent invasive mechanical ventilation owing to ARDS.

**Exposures:**

Patients in the full vaccination group had completed the primary COVID-19 vaccination series more than 14 days but less than 5 months prior to intubation. This time threshold was chosen because guidelines from the US Centers for Disease Control and Prevention recommend a booster dose beyond that time. The remaining patients (ie, those who were unvaccinated, partially vaccinated, or fully vaccinated <14 days or >5 months before intubation) comprised the control group.

**Main Outcomes and Measures:**

The primary outcome was time from intubation to all-cause intensive care unit mortality. A Cox proportional hazards regression model including vaccination status, age, comorbid conditions, and baseline Sequential Organ Failure Assessment score on the day of intubation was used.

**Results:**

A total of 265 intubated patients (170 men [64.2%]; median age, 66.0 years [IQR, 58.0-76.0 years]; 26 [9.8%] in the full vaccination group) were included in the study. A total of 20 patients (76.9%) in the full vaccination group received the BNT162b2 vaccine, and the remaining 6 (23.1%) received the ChAdOx1 nCoV-19 vaccine. Patients in the full vaccination group were older (median age, 72.5 years [IQR, 62.8-80.0 years] vs 66.0 years [IQR, 57.0-75.0 years]) and more likely to have comorbid conditions (24 of 26 [92.3%] vs 160 of 239 [66.9%]), including malignant neoplasm (6 of 26 [23.1%] vs 18 of 239 [7.5%]), than those in the control group. Full vaccination status was significantly associated with lower mortality compared with controls (16 of 26 patients [61.5%] died in the full vaccination group vs 163 of 239 [68.2%] in the control group; hazard ratio, 0.55 [95% CI, 0.32-0.94]; *P* = .03).

**Conclusions and Relevance:**

In this cohort study, full vaccination status was associated with lower mortality compared with controls, which suggests that vaccination might be beneficial even among patients who were intubated owing to COVID-19–related ARDS. These results may inform discussions with families about prognosis.

## Key Points

**Question:**

Is there an association between COVID-19 vaccination status and mortality among critically ill patients who require invasive mechanical ventilation owing to acute respiratory distress syndrome related to COVID-19?

**Findings:**

In this multicenter cohort study comprising 265 patients, after adjustment for confounders, full vaccination status compared with controls was associated with lower mortality among critically ill patients who required invasive mechanical ventilation owing to COVID-19–related acute respiratory distress syndrome.

**Meaning:**

The findings of this study suggest that full vaccination may be associated with lower mortality among patients who were intubated owing to COVID-19–related acute respiratory distress syndrome; therefore, total benefits of vaccination against COVID-19 may exceed those estimated from the prevention of invasive mechanical ventilation alone.

## Teaser

This multicenter cohort study examines the association between COVID-19 vaccination status and mortality among critically ill patients who require invasive mechanical ventilation owing to acute respiratory distress syndrome (ARDS) related to COVID-19.

## Introduction

Vaccination against COVID-19 is highly effective for preventing both documented SARS-CoV-2 infection and symptomatic disease.^1^ Nevertheless, despite full vaccination, SARS-CoV-2 infections (ie, breakthrough infections) do occur and are anticipated to increase in prevalence after the increasing vaccine coverage of the population and the emergence of new variants with increasing capacity for immune escape.^2,3^ Therefore, a comprehensive interpretation of the protective benefits associated with vaccination must account for prognosis after breakthrough infections.^4^

There is evidence that vaccinated people with breakthrough infections, as opposed to unvaccinated people, are less likely to develop severe symptoms (if any) and are less likely to require hospitalization.^5^ Even if they require hospitalization for COVID-19, vaccinated patients are less likely than unvaccinated patients to develop acute respiratory distress syndrome (ARDS) and progress to invasive mechanical ventilation.^6^ However, it is not yet clear whether vaccinated patients who progress to invasive mechanical ventilation still have a better prognosis than unvaccinated patients. This information is clinically relevant to inform discussions with families about the prognosis of their critically ill relatives who required invasive mechanical ventilation despite their prior full vaccination status. To expand our understanding of the benefits associated with COVID-19 vaccination, we examined the association between vaccination status and mortality among critically ill patients who required invasive mechanical ventilation owing to ARDS related to COVID-19.

## Methods

### Study Design

We performed a multicenter, observational retrospective cohort study including consecutive adult patients (aged >18 years) with polymerase chain reaction–confirmed SARS-CoV-2 infection who received invasive mechanical ventilation owing to ARDS during their hospitalization in an academic intensive care unit (ICU) at 3 tertiary hospitals in Athens, Greece (recruitment period from July 1 to November 29, 2021); Ioannina, Greece (recruitment period from September 25 to November 30, 2021); and Larissa, Greece (recruitment period from June 7, 2021, to February 1, 2022). The institutional review board at each participating study site (Athens: Evangelismos Hospital, Ioannina: University Hospital of Ioannina, and Larissa: University Hospital of Larissa) approved of the data collection and waived the need of informed consent owing to the observational study design and the collection of deidentified data. This study followed the Strengthening the Reporting of Observational Studies in Epidemiology (STROBE) reporting guideline.

### Data Collection and Study Groups

We collected information on age, sex, race and ethnicity (taken from the patients’ medical records), comorbid conditions (including malignant neoplasms), and Sequential Organ Failure Assessment (SOFA) score on the day of intubation (the respiratory component of the SOFA score was calculated after the intubation, while the remaining SOFA score components [ie, coagulation, hepatic, cardiovascular, neurologic, and kidney] were calculated prior to intubation) along with use (and its duration) of high-flow nasal oxygen and nonrebreather mask prior to intubation. We also collected data on ventilator settings, lung mechanics, arterial blood gas values, and severity of ARDS (determined according to the Berlin Definition^7^) on the day of intubation and on the third day after intubation, along with the outcomes of the patients included in each study group.

We categorized the included patients into 2 study groups. Patients in the full vaccination group had completed their primary COVID-19 vaccination series more than 14 days but less than 5 months prior to intubation. We chose this time threshold (ie, 5 months) because guidelines from the US Centers for Disease Control and Prevention recommend a booster dose beyond that time.^8^ The remaining patients (ie, unvaccinated, partially vaccinated, or fully vaccinated <14 days or >5 months prior to intubation) comprised the control group.

For each patient, we determined vaccination status, type of administered vaccine (if any), and time since vaccination using a national prescription platform. Outside this platform, no medication (including vaccines) could be prescribed.

### Study Outcomes

The primary outcome of this study was time from intubation to all-cause ICU mortality. Secondary outcomes were time from intubation to all-cause 28-day mortality, time from intubation to all-cause in-hospital mortality, length of ICU stay among survivors, length of hospital stay among survivors, occurrence of bacteremia among survivors, use of vasopressors, number of vasopressor-free days, use of continuous kidney replacement therapy (CKRT), number of CKRT-free days, number of ventilator-free days, and number of ICU-free days. All outcomes, apart from mortality and length of stay, were censored at day 28 after intubation. Patients discharged from the ICU with unassisted breathing before 28 days were considered to be alive at 28 days without needing vasopressors or CKRT.^9,10^ The number of vasopressor-free days, CKRT-free days, ventilator-free days, and ICU-free days was calculated by the number of days in the first 28 days after intubation that a patient was alive and not receiving vasopressors, not receiving CKRT, not receiving mechanical ventilation, or not in the ICU, respectively.^11,12^ The number of ICU-free days was also assessed at 60 days after intubation.

### Statistical Analysis

In this retrospective study, we included patients consecutively admitted to the participating ICUs during the study period without a priori sample size calculation. We presented continuous variables as median (IQR) values and compared them using the Mann-Whitney test (if 2 groups) or the Kruskal-Wallis test (if multiple groups). We presented categorical variables as frequencies and percentages and compared them using the χ^2^ test or the Fisher exact test, as appropriate. For time from intubation to all-cause mortality (ie, ICU mortality, 28-day mortality, and in-hospital mortality), we used a Cox proportional hazards regression model including vaccination status (full vaccination vs control), age, comorbid conditions, and baseline SOFA score on the day of intubation; we presented relevant results as hazard ratios (HRs) and 95% CIs, and we plotted the corresponding Cox-generated estimated survival curves.

For the primary outcome (ie, time from intubation to all-cause ICU mortality), we performed sensitivity analyses (1) by including only patients receiving an mRNA vaccine (BNT162b2) in the full vaccination group; (2) by including only unvaccinated patients in the control group; (3) by separating the control group into unvaccinated and remotely vaccinated patients (ie, those vaccinated >5 months before intubation); and (4) by expanding the aforementioned Cox proportional hazards regression model to also include severe ARDS.^7^

To construct the Cox proportional hazards regression models, we used all available information on mortality outcomes and the included variables (ie, vaccination status, age, comorbid conditions, and baseline SOFA score on the day of intubation). Data on baseline SOFA score were missing for 2 patients and were not imputed. All *P* values were from 2-sided tests, and results were deemed statistically significant at *P* < .05. We performed statistical analyses using SPSS software, version 28.0 (SPSS Inc).

## Results

A total of 265 intubated patients (170 men [64.2%] and 95 women [35.8%]; median age, 66.0 years [IQR, 58.0-76.0 years]) who underwent invasive mechanical ventilation owing to ARDS associated with COVID-19 were included in our study. eTable 1 in the Supplement shows the baseline characteristics, lung mechanics on the day of intubation, vaccination status, and outcomes of the included patients across the 3 participating sites (ie, Athens [103 patients], Ioannina [42 patients], and Larissa [120 patients]).

### Baseline Characteristics and Lung Mechanics of Patients in Each Study Group

Table 1 shows the baseline characteristics of the patients included in each study group.^7^ Of the 26 patients included in the full vaccination group, 20 (76.9%) received the BNT162b2 vaccine (Pfizer BioNTech), and the remaining 6 (23.1%) received the ChAdOx1 nCoV-19 vaccine (AstraZeneca). Patients in the full vaccination group were older (median age, 72.5 years [IQR, 62.8-80.0 years] vs 66.0 years [IQR, 57.0-75.0 years]) and more likely to have comorbid conditions (24 of 26 patients [92.3%] vs 160 of 239 patients [66.9%]), such as malignant neoplasm (6 of 26 patients [23.1%] vs 18 of 239 patients [7.5%]), than those in the control group. There were no substantial differences between groups in terms of sex, race and ethnicity, duration from symptom onset to intubation, and use (and its duration) of high-flow nasal oxygen and a nonrebreather mask. Similarly, there were no substantial differences between groups in terms of SOFA score on the day of intubation. Table 1 also shows the lung mechanics of the patients included in each study group.^7^ At baseline, patients in the full vaccination group had lower plateau pressure (median, 24.0 cm H_2_O [IQR, 21.0-28.0 cm H_2_O] vs 26.0 cm H_2_O [IQR, 24.0-29.0 cm H_2_O]), had higher oxygenation (assessed by the partial pressure of arterial oxygen to fraction of inspired oxygen ratio [Pao_2_:FiO_2_]; median, 119.0 [IQR, 77.2-206.3] vs median, 87.5 [IQR, 66.0-138.5]), and were less likely to have severe ARDS (11 of 25 patients [44.0%] vs 137 of 236 patients [58.1%]) compared with the control group, although the comparisons did not reach statistical significance. On the third day after intubation, the median Pao_2_:FiO_2_ was 180.0 (IQR, 107.0-217.0) in the full vaccination group vs 158.0 (IQR, 113.8-200.0) in the control group.

**Table 1.** Baseline Characteristics and Lung Mechanics of Patients in Each Study Group

Characteristic	All (N = 265)^a^	Full vaccination group (n = 26)	Control group (n = 239)	*P* value
Age, median (IQR), y	66.0 (58.0-76.0)	72.5 (62.8-80.0)	66.0 (57.0-75.0)	.04
Sex, No. (%)				
Female	95 (35.8)	9 (34.6)	86 (36.0)	.89
Male	170 (64.2)	17 (65.4)	153 (64.0)	
Race and ethnicity, No. (%)				
White	264 (99.6)	26 (100)	238 (99.6)	>.99
Other^b^	1 (0.4)	0	1 (0.4)	
Comorbid conditions, No. (%)	184 (69.4)	24 (92.3)	160 (66.9)	.008
Chronic kidney disease	18 (6.8)	5 (19.2)	13 (5.4)	.02
Chronic lung disease	22 (8.3)	3 (11.5)	19 (7.9)	.46
Heart condition^c^	50 (18.9)	9 (34.6)	41 (17.2)	.06
Hypertension	135 (50.9)	18 (69.2)	117 (49.0)	.05
Liver disease	2 (0.8)	1 (3.8)	1 (0.4)	.19
Diabetes	57 (21.5)	6 (23.1)	51 (21.3)	.84
Malignant neoplasm	24 (9.1)	6 (23.1)	18 (7.5)	.02
Autoimmune disorder	17 (6.4)	5 (19.2)	12 (5.0)	.02
Duration from symptom onset to intubation, median (IQR), d	12.0 (9.0-15.0)	12.0 (8.0-16.3)	12.0 (9.0-15.0)	.87
Use of high-flow nasal oxygen prior to intubation, No. (%)	205 (77.4)	22 (84.6)	183 (76.6)	.41
Duration of high-flow nasal oxygen prior to intubation, median (IQR), d	2.0 (1.0-5.0)	2.0 (1.0-5.0)	2.0 (1.0-5.0)	.57
Use of nonrebreather mask prior to intubation, No. (%)	125 (47.2)	13 (50.0)	112 (46.9)	.79
Duration of nonrebreather mask prior to intubation, median (IQR), d	2.0 (1.0-4.0)	2.0 (1.5-2.5)	2.0 (1.0-4.0)	.59
SOFA score on day of intubation, median (IQR)	5.0 (4.0-7.0)	5.0 (4.0-7.0)	5.0 (4.0-7.0)	.88
Respiratory	4.0 (3.0-4.0)	4.0 (2.5-4.0)	4.0 (3.0-4.0)	.07
Coagulation	0	0.0 (0.0-0.5)	0	.07
Hepatic	0	0	0	.18
Cardiovascular	1.0 (0.0-2.0)	0.0 (0.0-1.0)	1.0 (0.0-2.0)	.14
Neurologic	0.0 (0.0-1.0)	0.0 (0.0-1.0)	0	.04
Kidney	0.0 (0.0-1.0)	0.0 (0.0-1.0)	0.0 (0.0-1.0)	.59
Lung mechanics on day of intubation				
Volume-controlled ventilation, No. (%)	265 (100)	26 (100)	239 (100)	NA
Respiratory rate, median (IQR), breaths/min	24.0 (21.0-26.0)	24.0 (22.0-26.0)	24.0 (21.0-26.0)	.75
Tidal volume, median (IQR), mL	440.0 (400.0-470.0)	450.0 (420.0-470.0)	440.0 (400.0-470.0)	.35
External PEEP, median (IQR), cm H_2_O	12.0 (10.0-13.0)	10.0 (10.0-12.0)	12.0 (10.0-13.3)	.12
Total PEEP, median (IQR), cm H_2_O	12.0 (10.0-13.0)	11.0 (10.0-12.0)	12.0 (10.0-14.0)	.11
Plateau pressure, median (IQR), cm H_2_O	26.0 (24.0-29.0)	24.0 (21.0-28.0)	26.0 (24.0-29.0)	.06
Driving pressure, median (IQR), cm H_2_O	14.0 (11.0-17.0)	14.0 (11.0-16.0)	14.0 (11.5-17.0)	.35
FiO_2_, median (IQR), %	1.0 (0.9-1.0)	1.0 (1.0-1.0)	1.0 (0.9-1.0)	.95
Pao_2_, median (IQR), mm Hg	85.0 (65.0-114.5)	107.0 (77.2-137.0)	85.0 (65.0-113.0)	.03
Pao_2_:FiO_2_, median (IQR)	88.0 (66.5-139.5)	119.0 (77.2-206.3)	87.5 (66.0-138.5)	.05
Paco_2_, median (IQR), mm Hg	49.0 (42.0-60.5)	45.0 (40.0-55.5)	49.0 (42.0-61.0)	.22
Severity of ARDS, No. (%)^d^				
Mild or moderate	113/261 (43.3)	14/25 (56.0)	99/236 (41.9)	.18
Severe	148/261 (56.7)	11/25 (44.0)	137/236 (58.1)	
Lung mechanics on 3rd day after intubation				
Still intubated or dead, No. (%)	262 (98.9)	25 (96.2)	237 (99.2)	.27
Total PEEP, median (IQR), cm H_2_O	11.0 (9.0-13.0)	10.0 (9.3-12.0)	11.0 (9.0-13.0)	.44
Plateau pressure, median (IQR), cm H_2_O	25.0 (22.0-27.0)	23.5 (20.3-25.8)	25.0 (22.0-27.5)	.17
Driving pressure, median (IQR), cm H_2_O	13.0 (11.0-15.0)	13.5 (10.3-14.8)	13.0 (11.0-15.0)	.35
Pao_2_:FiO_2_, median (IQR)	160.0 (111.0-200.0)	180.0 (107.0-217.0)	158.0 (113.8-200.0)	.56

Abbreviations: ARDS, acute respiratory distress syndrome; FiO_2_, fraction of inspired oxygen; NA, not applicable; Paco_2_, partial pressure of arterial carbon dioxide; Pao_2_, partial pressure of arterial oxygen; PEEP, positive end expiratory pressure; SOFA, Sequential Organ Failure Assessment.

^a^ Of the 26 patients included in the full vaccination group, 20 (76.9%) received the BNT162b2 vaccine (Pfizer BioNTech; 3 of those patients also received a third dose), and the remaining 6 (23.1%) received the ChAdOx1 nCoV-19 vaccine (AstraZeneca; no patient received a third dose). Of the 239 patients included in the control group, 206 (86.2%) were unvaccinated, 32 (13.4%) were remotely vaccinated (ie, >5 months before intubation), and 1 (0.4%) was vaccinated less than 14 days before intubation. Of the 33 vaccinated patients in the control group, 25 (75.8%) received the BNT162b2 vaccine (Pfizer BioNTech), 3 (9.1%) received the mRNA-1273 vaccine (Moderna), and 5 (15.2%) received the ChAdOx1 nCoV-19 vaccine (AstraZeneca) vaccine.

^b^ Included self-defined Roma.

^c^ Included congestive heart failure, coronary artery disease, and cardiomyopathies.

^d^ Data on severity of ARDS were missing for 4 patients (1 in the full vaccination group and 3 in the control group). Severity of ARDS was determined according to the Berlin Definition.^7^

### Outcomes of Patients in Each Study Group

**Figure 1.**
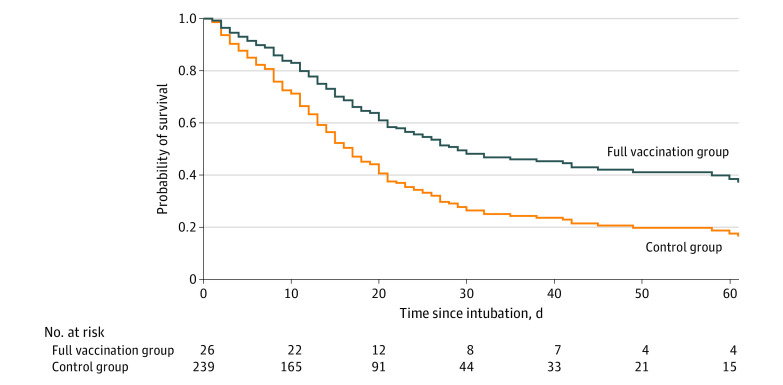
Survival Curves of Patients Included in the Full Vaccination and Control Groups For time-to-event analysis from intubation to all-cause intensive care unit mortality, we used a Cox proportional hazards regression model including vaccination status (full vaccination vs control), age, comorbid conditions, and baseline Sequential Organ Failure Assessment score on the day of intubation and plotted the corresponding Cox-generated estimated survival curves. Full vaccination status was significantly associated with lower mortality compared with controls (hazard ratio, 0.55 [95% CI, 0.32-0.94]; *P* = .03; relative reduction, 45%).

Table 2 shows a Cox proportional hazards regression model to isolate the association of vaccination status (full vaccination vs control), age, comorbid conditions, and SOFA score on the day of intubation with all-cause ICU mortality. Full vaccination status was significantly associated with lower mortality compared with controls (16 of 26 patients [61.5%] in the full vaccination group died vs 163 of 239 [68.2%] in the control group; HR, 0.55 [95% CI, 0.32-0.94]; *P* = .03; relative reduction, 45%). Figure 1 shows the corresponding survival curves for each group.

**Table 2.** Cox Proportional Hazards Regression Model^a^

Variable	Hazard ratio (95% CI)	*P* value
Full vaccination status (vs control)	0.55 (0.32-0.94)	.03
Age (increments of 1 y)	1.02 (1.01-1.03)	.007
Comorbid conditions (any vs none)	1.30 (0.89-1.91)	.17
SOFA score (increments of 1)	1.22 (1.14-1.29)	<.001

Abbreviation: SOFA, Sequential Organ Failure Assessment.

^a^ To isolate the association of vaccination status, age, comorbid conditions, and SOFA score on the day of intubation with all-cause mortality.

**Figure 2.**
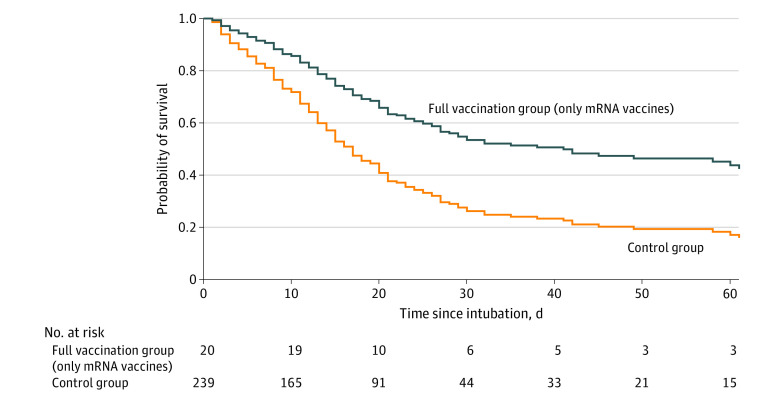
Survival Curves of Patients Included in the Full Vaccination and Control Groups in the Sensitivity Analysis by Including Only Patients Receiving mRNA Vaccine For time-to-event analysis from intubation to all-cause intensive care unit mortality, we used a Cox proportional hazards regression model including vaccination status (full vaccination vs control), age, comorbid conditions, and baseline Sequential Organ Failure Assessment score on the day of intubation and plotted the corresponding Cox-generated estimated survival curves. Full vaccination status was significantly associated with lower mortality compared with controls (hazard ratio, 0.47 [95% CI, 0.25-0.87]; *P* = .02).

Similarly, full vaccination status was associated with lower mortality compared with controls in the sensitivity analyses (1) by including only patients receiving an mRNA vaccine in the full vaccination group (HR, 0.47 [95% CI, 0.25-0.87]; *P* = .02) (eTable 2 in the Supplement; Figure 2); (2) by including only unvaccinated patients in the control group (HR, 0.54 [95% CI, 0.31-0.94]; *P* = .03) (eTable 3 in the Supplement); (3) by separating the control group into unvaccinated and remotely vaccinated patients (ie, those vaccinated >5 months before intubation) (HR, 0.54 [95% CI, 0.31-0.93]; *P* = .03) (eTable 4 and eFigure 1 in the Supplement); and (4) by expanding the aforementioned Cox proportional hazards regression model to also include severe ARDS (HR, 0.58 [95% CI, 0.34-1.00]; *P* = .05) (eTable 5 in the Supplement).

With regard to secondary outcomes, in Cox proportional hazards regression models adjusting for age, comorbid conditions, and SOFA score on the day of intubation, the HR for the association between full vaccination vs control and 28-day mortality was 0.40 (95% CI, 0.21-0.75; *P* = .004) (eTable 6 in the Supplement), while the HR for the association between full vaccination vs control and in-hospital mortality was 0.63 (95% CI, 0.38-1.03; *P* = .07) (eTable 7 and eFigure 2 in the Supplement). Table 3 shows the additional secondary outcomes of patients in each study group. There were no statistically significant differences between study groups in terms of length of ICU stay among survivors, length of hospital stay among survivors, occurrence of bacteremia among survivors, use of vasopressors, number of vasopressor-free days, use of CKRT, number of CKRT-free days, number of ventilator-free days, and number of ICU-free days.

**Table 3.** Secondary Outcomes of Patients in Each Study Group

Outcome^a^	Median (IQR) value			*P* value
	All (N = 265)^b^	Full vaccination group (n = 26)	Control group (n = 239)	
Length of ICU stay among survivors, d	27.0 (16.0-50.0)	20.0 (10.0-70.0)	27.5 (16.0-45.8)	.87
Length of hospital stay among survivors, d	38.0 (28.0-70.0)	70.0 (36.0-83.0)	36.5 (27.3-60.8)	.11
Occurrence of bacteremia among survivors, No. (%)	73/102 (71.6)	8/14 (57.1)	65/88 (73.9)	.21
Use of vasopressors, No. (%)	262 (98.9)	25 (96.2)	237 (99.2)	>.99
No. of vasopressor-free days	0.0 (0.0-8.0)	1.0 (0.0-11.5)	0.0 (0.0-8.0)	.29
No. of vasopressor-free days, mean (SD)	5.2 (7.9)	6.4 (9.2)	5.1 (7.7)	.29
Use of continuous kidney replacement therapy, No. (%)	97 (36.6)	9 (34.6)	88 (36.8)	.92
No. of continuous kidney replacement therapy–free days	14.0 (5.0-28.0)	19.0 (6.0-28.0)	13.0 (5.0-28.0)	.23
No. of ventilator-free days	0	0.0 (0.0-10.0)	0	.16
No. of ventilator-free days, mean (SD)	3.3 (6.9)	5.0 (8.5)	3.1 (6.7)	.16
No. of ICU-free days^c^	0	0	0	.59
No. of ICU-free days, mean (SD)	1.9 (5.0)	2.4 (5.8)	1.8 (5.0)	.59

Abbreviation: ICU, intensive care unit.

^a^ All outcomes apart from length of stay were censored at day 28 after intubation. Patients discharged from the ICU with unassisted breathing before 28 days were considered to be alive at 28 days without needing vasopressors or continuous kidney replacement therapy. The number of vasopressor-free days, continuous kidney replacement therapy–free days, ventilator-free days, and ICU-free days was calculated by the number of days in the first 28 days after intubation that a patient was alive and not receiving vasopressors, not receiving continuous kidney replacement therapy, not receiving mechanical ventilation, or not in the ICU, respectively.

^b^ For 2 patients (1 in the full vaccination group and 1 in the control group), relevant data were missing.

^c^ There was no difference between the full vaccination group (0.0 [0.0-7.3] days) and the control group (0.0 [0.0-8.0] days) in terms of 60-day ICU-free days (*P* = .92).

## Discussion

By incorporating data from 265 intubated patients who underwent invasive mechanical ventilation owing to COVID-19–related ARDS, we found that patients in the full vaccination group were older and more likely to have comorbid conditions but, after adjustment for confounders, were less likely to die (HR, 0.55 [95% CI, 0.32-0.94]) compared with patients in the control group. These findings suggest that the total benefits associated with vaccination against COVID-19 may exceed those previously estimated from the prevention of invasive mechanical ventilation alone.

We found that patients in the full vaccination group were older and more likely to have comorbid conditions, including malignant neoplasm, suggesting an impaired immune status. This finding is in line with evidence that the association between vaccination and reduced risk of hospitalization due to COVID-19 is substantially weaker for patients with impaired immune status.^6^ For example, a large observational study involving 4513 hospitalized adults in 21 US hospitals showed that the benefit associated with mRNA vaccination against hospitalization for COVID-19 was lower for immunocompromised than immunocompetent patients.^6^ Such evidence may support recommendations for booster vaccine doses, especially for patients with impaired immune status.^13^

When analyzing our data, we took into consideration the time since vaccination. We separated patients into those who had completed their primary COVID-19 vaccination series more than 14 days but less than 5 months prior to intubation (full vaccination group) vs the remaining patients (control group, including unvaccinated, partially vaccinated, or fully vaccinated individuals <14 days or >5 months prior to intubation). Moreover, we confirmed the time since vaccination (along with vaccination status itself) using a national prescription platform (rather than self-report); this accuracy of vaccination data might minimize any misclassification bias and therefore may enhance the reliability of our results. Our choice to take time since vaccination into consideration was based on several previous studies indicating that protection against infection from vaccination (specifically with mRNA vaccines, such as BNT162b2, which was administered to 76.9% of patients in the full vaccination group) may decrease over time.^5,14,15^ Our study may contribute to this growing body of evidence by focusing on critically ill patients with COVID-19 who eventually underwent invasive mechanical ventilation. The results of our sensitivity analysis that separated the control group into unvaccinated and remotely vaccinated patients (ie, those vaccinated >5 months before intubation) (eFigure 1 in the Supplement) may corroborate the decrease in the effectiveness of vaccination over time.

We found that, among patients who underwent invasive mechanical ventilation owing to COVID-19–related ARDS, full vaccination was associated with lower mortality compared with controls after adjusting for confounders. An explanation of this finding is hindered by the small number of patients in the full vaccination group, which may only allow us to make conjectures. One conjecture is that full vaccination, even when it fails to fully prevent the development of ARDS, may still attenuate its severity. This conjecture is supported by the observation that patients in the full vaccination group had better (albeit statistically nonsignificant) lung mechanics and higher oxygenation (ie, less severe ARDS) at baseline than patients in the control group. Even on the third day after intubation, oxygenation was higher in the full vaccination group than in the control group. This finding is important because evidence both before and during the COVID-19 era showed that, among patients with ARDS, oxygenation on the third day after intubation may be more strongly associated with mortality than oxygenation on the day of intubation.^16,17^ Also, the occurrence of bacteremia among survivors was 57.1% (8 of 14) in the full vaccination group vs 73.9% (65 of 88) in the control group, and, although statistically insignificant, this difference in occurrence might be associated with different mortality rates between groups.^18^

### Limitations

Our study has some limitations. First, the full vaccination group included 26 patients. Although the fact that this number was small might be not surprising given that vaccination prevents severe illness requiring intubation,^6^ it did not allow for stable estimates of the characteristics for the full vaccination group. Also, this number did not allow for the performance of direct comparisons of the effectiveness of different vaccine doses (2 vs 3) or different vaccine types among our population of critically ill patients requiring intubation, although such differences between vaccine types have been found in the general population of patients with COVID-19.^19,20^ However, we performed a sensitivity analysis by including only patients in the full vaccination group receiving an mRNA vaccine and found that full vaccination status was associated with lower mortality compared with controls (Figure 2). Second, we could not rule out the presence of selection bias given that we lacked data on the number of patients (if any) with COVID-19 who were not offered intubation and/or ICU admission at the participating study sites. However, we speculate that this number would probably be negligible on the basis of evidence that withholding intubation is not a common practice among physicians in Greece and that the criteria for ICU admission were not strict during the COVID-19 era.^21,22^

Third, given the observational cohort design of our study, we could not rule out residual confounding (especially given the difference in the number of patients included in each study group). However, we attempted to control for several relevant confounders, such as age, comorbid conditions, and baseline disease severity, along with time since vaccination. Moreover, despite their inevitable disadvantage from residual confounding, observational studies involving hospitalized patients with several comorbid conditions may offer the advantage that they are more representative of the inpatient population than randomized clinical trials involving the general population.^6^ Fourth, in light of the evidence regarding the potential decreased effectiveness of vaccination against the SARS-CoV-2 Delta (B.1.617.2) variant,^23,24^ our lack of data on viral sequencing may be considered a limitation. However, similar to several previous analyses,^15,25,26^ we may use the period of study recruitment as a proxy for Delta variant infections. Given that the Delta variant was the dominant variant in Greece when most of our patients were recruited (fall of 2021), we may safely assume that most of our intubated patients were probably infected with the Delta variant. However, a recent study asserted that attenuation of vaccine effectiveness associated with variants may not be as great as that associated with time since vaccination^15^; the latter parameter was taken into consideration in our study.

## Conclusions

This cohort study found that full vaccination status was associated with lower mortality compared with controls, which suggests that vaccination might be beneficial even among patients who were intubated owing to COVID-19–related ARDS. By showing that the total benefits associated with vaccination against COVID-19 may exceed those estimated from the prevention of invasive mechanical ventilation alone, these results expand our understanding of the outcomes of patients with breakthrough infections. This knowledge may be useful given the anticipated, ever-increasing prevalence of such infections and may inform discussions with families about the prognosis of intubated patients.^27^

## Notes

Supplement. **eTable 1.** Baseline Characteristics, Lung Mechanics on the Day of Intubation, Vaccination Status and Outcomes of Included Patients Across the Three Participating Study Sites **eTable 2.** Cox Proportional Hazards Regression Model to Isolate the Contribution of Vaccination Status, Age, Comorbidity and SOFA Score on the Day of Intubation (Independent Variables) to All-Cause Intensive Care Unit Mortality (Dependent Variable) in the Sensitivity Analysis by Including Only Patients Receiving mRNA Vaccine **eTable 3.** Cox Proportional Hazards Regression Model to Isolate the Contribution of Vaccination Status, Age, Comorbidity and SOFA Score on the Day of Intubation (Independent Variables) to All-Cause Intensive Care Unit Mortality (Dependent Variable) in the Sensitivity Analysis by Including Only Unvaccinated Patients in the Control Group **eTable 4.** Cox Proportional Hazards Regression Model to Isolate the Contribution of Vaccination Status, Age, Comorbidity and SOFA Score on the Day of Intubation (Independent Variables) to All-Cause Intensive Care Unit Mortality (Dependent Variable) in the Sensitivity Analysis by Separating the Control Group Into Unvaccinated and Remotely Vaccinated Patients (i.e., Those Vaccinated More Than Five Months Before Intubation) **eFigure 1.** Survival Curves in the Sensitivity Analysis by Separating the Control Group Into Unvaccinated and Remotely Vaccinated Patients (i.e., Those Vaccinated More Than Five Months Before Intubation) **eTable 5.** Cox Proportional Hazards Regression Model to Isolate the Contribution of Vaccination Status, Age, Comorbidity, SOFA Score, and Presence of Severe ARDS on the Day of Intubation (Independent Variables) to All-Cause Intensive Care Unit Mortality (Dependent Variable) **eTable 6.** Cox Proportional Hazards Regression Model to Isolate the Contribution of Vaccination Status, Age, Comorbidity, and SOFA Score on the Day of Intubation (Independent Variables) to All-Cause Mortality by Day 28 Following Intubation (Dependent Variable) **eTable 7.** Cox Proportional Hazards Regression Model to Isolate the Contribution of Vaccination Status, Age, Comorbidity, and SOFA Score on the Day of Intubation (Independent Variables) to All-Cause In-Hospital Mortality (Dependent Variable) **eFigure 2.** Survival Curves of Patients Included in the Full Vaccination vs Control Group
