# Supplementary material for: Association Between Vaccination Status and Mortality Among Intubated Patients With COVID-19–Related Acute Respiratory Distress Syndrome
Source: JAMA Netw Open. 2022 Oct 7;5(10):e2235219. doi: 10.1001/jamanetworkopen.2022.35219 (PMC9547321; doi:10.1001/jamanetworkopen.2022.35219)
Supplement: Supplement. — eTable 1. Baseline Characteristics, Lung Mechanics on the Day of Intubation, Vaccination Status and Outcomes of Included Patients Across the Three Participating Study Sites eTable 2. Cox Proportional Hazards Regression Model to Isolate the Contribution of Vaccination Status, Age, Comorbidity and SOFA Score on the Day of Intubation (Independent Variables) to All-Cause Intensive Care Unit Mortality (Dependent Variable) in the Sensitivity Analysis by Including Only Patients Receiving mRNA Vaccine eTable 3. Cox Proportional Hazards Regression Model to Isolate the Contribution of Vaccination Status, Age, Comorbidity and SOFA Score on the Day of Intubation (Independent Variables) to All-Cause Intensive Care Unit Mortality (Dependent Variable) in the Sensitivity Analysis by Including Only Unvaccinated Patients in the Control Group eTable 4. Cox Proportional Hazards Regression Model to Isolate the Contribution of Vaccination Status, Age, Comorbidity and SOFA Score on the Day of Intubation (Independent Variables) to All-Cause Intensive Care Unit Mortality (Dependent Variable) in the Sensitivity Analysis by Separating the Control Group Into Unvaccinated and Remotely Vaccinated Patients (i.e., Those Vaccinated More Than Five Months Before Intubation) eFigure 1. Survival Curves in the Sensitivity Analysis by Separating the Control Group Into Unvaccinated and Remotely Vaccinated Patients (i.e., Those Vaccinated More Than Five Months Before Intubation) eTable 5. Cox Proportional Hazards Regression Model to Isolate the Contribution of Vaccination Status, Age, Comorbidity, SOFA Score, and Presence of Severe ARDS on the Day of Intubation (Independent Variables) to All-Cause Intensive Care Unit Mortality (Dependent Variable) eTable 6. Cox Proportional Hazards Regression Model to Isolate the Contribution of Vaccination Status, Age, Comorbidity, and SOFA Score on the Day of Intubation (Independent Variables) to All-Cause Mortality by Day 28 Following Intubation (Dependent Variable) eTa [file jamanetwopen-e2235219-s001.pdf]

Supplementary Online Content

Grapsa E, Adamos G, Andrianopoulos I, et al. Association between vaccination status and mortality among intubated patients with COVID-19–related acute respiratory distress syndrome. *JAMA Netw Open.* 2022;5(10):e2235219.

doi:10.1001/jamanetworkopen.2022.35219

eTable 1. Baseline Characteristics, Lung Mechanics on the Day of Intubation, Vaccination Status and Outcomes of Included Patients Across the Three Participating Study Sites
eTable 2. Cox Proportional Hazards Regression Model to Isolate the Contribution of Vaccination Status, Age, Comorbidity and SOFA Score on the Day of Intubation (Independent Variables) to All-Cause Intensive Care Unit Mortality (Dependent Variable) in the Sensitivity Analysis by Including Only Patients Receiving mRNA Vaccine
eTable 3. Cox Proportional Hazards Regression Model to Isolate the Contribution of Vaccination Status, Age, Comorbidity and SOFA Score on the Day of Intubation (Independent Variables) to All-Cause Intensive Care Unit Mortality (Dependent Variable) in the Sensitivity Analysis by Including Only Unvaccinated Patients in the Control Group
eTable 4. Cox Proportional Hazards Regression Model to Isolate the Contribution of Vaccination Status, Age, Comorbidity and SOFA Score on the Day of Intubation (Independent Variables) to All-Cause Intensive Care Unit Mortality (Dependent Variable) in the Sensitivity Analysis by Separating the Control Group Into Unvaccinated and Remotely Vaccinated Patients (i.e., Those Vaccinated More Than Five Months Before Intubation)
eFigure 1. Survival Curves in the Sensitivity Analysis by Separating the Control Group Into Unvaccinated and Remotely Vaccinated Patients (i.e., Those Vaccinated More Than Five Months Before Intubation)
eTable 5. Cox Proportional Hazards Regression Model to Isolate the Contribution of Vaccination Status, Age, Comorbidity, SOFA Score, and Presence of Severe ARDS on the Day of Intubation (Independent Variables) to All-Cause Intensive Care Unit Mortality (Dependent Variable)
eTable 6. Cox Proportional Hazards Regression Model to Isolate the Contribution of Vaccination Status, Age, Comorbidity, and SOFA Score on the Day of Intubation (Independent Variables) to All-Cause Mortality by Day 28 Following Intubation (Dependent Variable)
eTable 7. Cox Proportional Hazards Regression Model to Isolate the Contribution of Vaccination Status, Age, Comorbidity, and SOFA Score on the Day of Intubation (Independent Variables) to All-Cause In-Hospital Mortality (Dependent Variable)
eFigure 2. Survival Curves of Patients Included in the Full Vaccination vs Control Group

This supplementary material has been provided by the authors to give readers additional information about their work.

© 2022 Grapsa E et al. *JAMA Network Open.*

eTable 1. Baseline Characteristics, Lung Mechanics on the Day of Intubation, Vaccination Status and Outcomes of Included Patients Across the Three Participating Study Sites

**Characteristic[a]**	**Total (n=265)**	**Athens (n=103)**	**Ioannina (n=42)**	**Larissa (n=120)**	**p value**
Age, years	66.0 (58.0-76.0)	69.0 (57.0-77.0)	69.5 (60.0-75.3)	64.5 (56.0-74.8)	0.28
Sex					0.29
Female	95 (35.8)	31 (30.1)	16 (38.1)	48 (40.0)	
Male	170 (64.2)	72 (69.9)	26 (61.9)	72 (60.0)	
Race					0.55
Caucasian	264 (99.6)	102 (99.0)	42 (100.0)	120 (100.0)	
Other[b]	1 (0.4)	1 (1.0)	0 (0.0)	0 (0.0)	
Comorbidity	184 (69.4)	73 (70.9)	26 (61.9)	85 (70.8)	0.51
Chronic kidney disease	18 (6.8)	10 (9.7)	2 (4.8)	6 (5.0)	0.32
Chronic lung disease	22 (8.3)	10 (9.7)	4 (9.5)	8 (6.7)	0.68
Heart condition[c]	50 (18.9)	30 (29.1)	3 (7.1)	17 (14.2)	0.002
Hypertension	135 (50.9)	49 (47.6)	20 (47.6)	66 (55.0)	0.49
Liver disease	2 (0.8)	2 (1.9)	0 (0.0)	0 (0.0)	0.44
Diabetes mellitus	57 (21.5)	22 (21.4)	9 (21.4)	26 (21.7)	1.0

© 2022 Grapsa E et al. *JAMA Network Open.*

Malignancy	24 (9.1)	16 (15.5)	1 (2.4)	7 (5.8)	0.01
Autoimmune	17 (6.4)	9 (8.7)	1 (2.4)	7 (5.8)	0.34
Duration from symptom onset to intubation, days	12.0 (9.0-15.0)	10.0 (6.0-14.0)	13.0 (7.0-16.0)	13.0 (11.0-16.0)	<0.001
Usage of high-flow nasal oxygen prior to intubation	206 (78.6)	85 (83.3)	25 (62.5)	96 (80.0)	0.02
Duration of high-flow nasal oxygen prior to intubation, days	4.0 (2.0-6.0)	2.0 (1.0-5.0)	4.0 (1.0-8.0)	4.0 (2.0-7.8)	0.001
Usage of non-rebreather mask prior to intubation	125 (47.5)	58 (56.3)	22 (55.0)	45 (37.5)	0.01
Duration of non-rebreather mask prior to intubation, days	2.0 (1.0-4.0)	1.0 (1.0-2.0)	3.0 (1.0-5.5)	3.0 (2.0-4.0)	<0.001
SOFA score on the day of intubation	5.0 (4.0-7.0)	6.0 (4.0-8.0)	4.0 (4.0-5.3)	5.0 (5.0-7.0)	<0.001
Respiratory	4.0 (3.0-4.0)	3.0 (3.0-4.0)	4.0 (3.0-4.0)	4.0 (4.0-4.0)	<0.001
Coagulation	0.0 (0.0-0.0)	0.0 (0.0-0.0)	0.0 (0.0-0.0)	0.0 (0.0-0.0)	0.44
Hepatic	0.0 (0.0-0.0)	0.0 (0.0-0.0)	0.0 (0.0-0.0)	0.0 (0.0-0.0)	0.05

© 2022 Grapsa E et al. *JAMA Network Open.*

Cardiovascular	1.0 (0.0-2.0)	0.0 (0.0-3.0)	0.0 (0.0-0.0)	1.0 (1.0-1.0)	<0.001
Neurologic	0.0 (0.0-1.0)	1.0 (0.0-1.0)	0.0 (0.0-0.0)	0.0 (0.0-0.0)	<0.001
Renal	0.0 (0.0-1.0)	0.0 (0.0-1.0)	0.0 (0.0-1.0)	0.0 (0.0-1.0)	0.38
**Lung mechanics on the day of intubation**					
Volume-controlled ventilation	265 (100.0)	103 (100.0)	42 (100.0)	120 (100.0)	-
Respiratory rate, bpm	24.0 (21.0-26.0)	25.0 (22.0-26.3)	20.0 (17.0-24.3)	25.0 (22.0-27.0)	<0.001
Tidal volume, mL	440.0 (400.0-470.0)	450.0 (420.0-480.0)	465.0 (440.0-502.5)	410.0 (352.5-450.0)	<0.001
PEEPext, cmH_2O	12.0 (10.0-13.0)	12.0 (10.0-14.0)	11.5 (10.0-13.0)	11.0 (10.0-12.0)	0.30
PEEPtotal, cmH_2O	12.0 (10.0-13.0)	12.0 (10.0-14.0)	11.5 (10.0-13.0)	11.0 (10.0-12.0)	0.16
Pplateau, cmH_2O	26.0 (24.0-29.0)	25.0 (22.0-28.0)	28.0 (25.0-30.3)	27.0 (24.0-29.0)	0.001
Pdriving, cmH_2O	14.0 (11.0-17.0)	12.0 (10.0-15.0)	17.0 (13.5-19.0)	14.0 (12.0-17.8)	<0.001
FiO_2	1.0 (0.9-1.0)	1.0 (0.9-1.0)	1.0 (0.8-1.0)	1.0 (1.0-1.0)	0.23
PaO_2, mmHg	85.0 (65.0-114.5)	117.0 (91.0-150.0)	75.5 (62.0-94.3)	72.0 (56.0-87.0)	<0.001
PaO_2:FiO_2	88.0 (66.5-139.5)	133.0 (97.0-180.0)	80.0 (65.8-137.8)	75.5 (56.0-90.0)	<0.001
$PaCO_2$, mmHg	49.0 (42.0-60.5)	45.0 (38.0-52.0)	43.0 (36.8-52.0)	56.0 (47.3-67.0)	<0.001

© 2022 Grapsa E et al. *JAMA Network Open.*

**Vaccination Status**					
Full	26 (9.8)	16 (15.5)	2 (4.8)	8 (6.7)	0.04
**Outcomes**[d,e]					
ICU-mortality	163 (61.5)	45 (43.7)	35 (83.3)	83 (69.2)	<0.001
Usage of vasopressors	262 (98.9)	100 (97.0)	42 (100.0)	120 (100.0)	0.54
Vasopressor-free days, days	0.0 (0.0-8.0)	2.0 (0.0-14.0)	0.0 (0.0-3.3)	0.0 (0.0-7.8)	0.002
Usage of continuous renal replacement therapy	97 (36.6)	34 (33.0)	6 (14.3)	57 (47.5)	<0.001
Continuous renal replacement therapy-free days, days	14.0 (5.0-28.0)	21.0 (5.5-28.0)	15.0 (8.0-23.0)	10.0 (3.0-27.5)	0.03
Ventilator-free days, days	0.0 (0.0-0.0)	0.0 (0.0-7.0)	0.0 (0.0-0.0)	0.0 (0.0-0.0)	0.003
ICU-free days, days	0.0 (0.0-0.0)	0.0 (0.0-0.0)	0.0 (0.0-0.0)	0.0 (0.0-0.0)	0.01

Abbreviations: n, number; SOFA, sequential organ failure assessment; bpm, breaths per minute; PEEP, positive end expiratory pressure; Pplateau, plateau pressure; Pdriving, driving pressure; PaO_2, partial pressure of arterial oxygen; FiO_2, fraction of inspired oxygen; $PaCO_2$, partial pressure of arterial carbon dioxide: ICU, intensive care unit.

© 2022 Grapsa E et al. *JAMA Network Open.*

[a]Data are presented as median (interquartile range) or number of patients (%).

[b]Other race included self-defined Roma.

[c]Heart condition included congestive heart failure, coronary artery disease, and cardiomyopathies.

[d]For two patients in Athens, data on secondary outcomes were missing.

[e]Outcomes were censored at day 28 following intubation. Patients discharged from ICU with unassisted breathing before 28 days were considered to be alive at 28 days without needing vasopressors or continuous renal replacement therapy. Vasopressor-free days, continuous renal replacement therapy-free days, ventilator-free days and ICU-free days were calculated by the number of days in the first 28 days following intubation that a patient was alive and not receiving vasopressors, not receiving continuous renal replacement therapy, not on a ventilator or not in the ICU, respectively.

© 2022 Grapsa E et al. *JAMA Network Open.*

eTable 2. Cox Proportional Hazards Regression Model to Isolate the Contribution of Vaccination Status, Age, Comorbidity and SOFA Score on the Day of Intubation (Independent Variables) to All-Cause Intensive Care Unit Mortality (Dependent Variable) in the Sensitivity Analysis by Including Only Patients Receiving mRNA Vaccine

	**Hazard ratio**	**95% confidence intervals**	**p value**
Full vaccination status (versus control)	0.47	0.25-0.87	0.02
Age (increments of 1)	1.02	1.01-1.03	0.007
Comorbidity (any versus none)	1.34	0.91-1.97	0.13
SOFA score (increments of 1)	1.21	1.14-1.29	<0.001

Abbreviations: SOFA, sequential organ failure assessment.

© 2022 Grapsa E et al. *JAMA Network Open.*

eTable 3. Cox Proportional Hazards Regression Model to Isolate the Contribution of Vaccination Status, Age, Comorbidity and SOFA Score on the Day of Intubation (Independent Variables) to All-Cause Intensive Care Unit Mortality (Dependent Variable) in the Sensitivity Analysis by Including Only Unvaccinated Patients in the Control Group

	**Hazard ratio**	**95% confidence intervals**	**p value**
Full vaccination status (versus control)	0.54	0.31-0.94	0.03
Age (increments of 1)	1.02	1.01-1.04	0.009
Comorbidity (any versus none)	1.31	0.89-1.93	0.17
SOFA score (increments of 1)	1.21	1.13-1.29	<0.001

Abbreviations: SOFA, sequential organ failure assessment.

© 2022 Grapsa E et al. *JAMA Network Open.*

eTable 4. Cox Proportional Hazards Regression Model to Isolate the Contribution of Vaccination Status, Age, Comorbidity and SOFA Score on the Day of Intubation (Independent Variables) to All-Cause Intensive Care Unit Mortality (Dependent Variable) in the Sensitivity Analysis by Separating the Control Group Into Unvaccinated and Remotely Vaccinated Patients (i.e., Those Vaccinated More Than Five Months Before Intubation)

	**Hazard ratio**	**95% confidence intervals**	**p value**
Vaccination status[a]			
Unvaccinated	Reference	Reference	Reference
Remotely vaccinated	0.89	0.56-1.40	0.61
Fully vaccinated	0.54	0.31-0.93	0.03
Age (increments of 1)	1.02	1.01-1.03	0.007
Comorbidity (any versus none)	1.33	0.90-1.95	0.15
SOFA score (increments of 1)	1.22	1.14-1.29	<0.001

Abbreviations: SOFA, sequential organ failure assessment.

[a]One patient was vaccinated less than 14 days before intubation and was excluded from this sensitivity analysis.

© 2022 Grapsa E et al. *JAMA Network Open.*

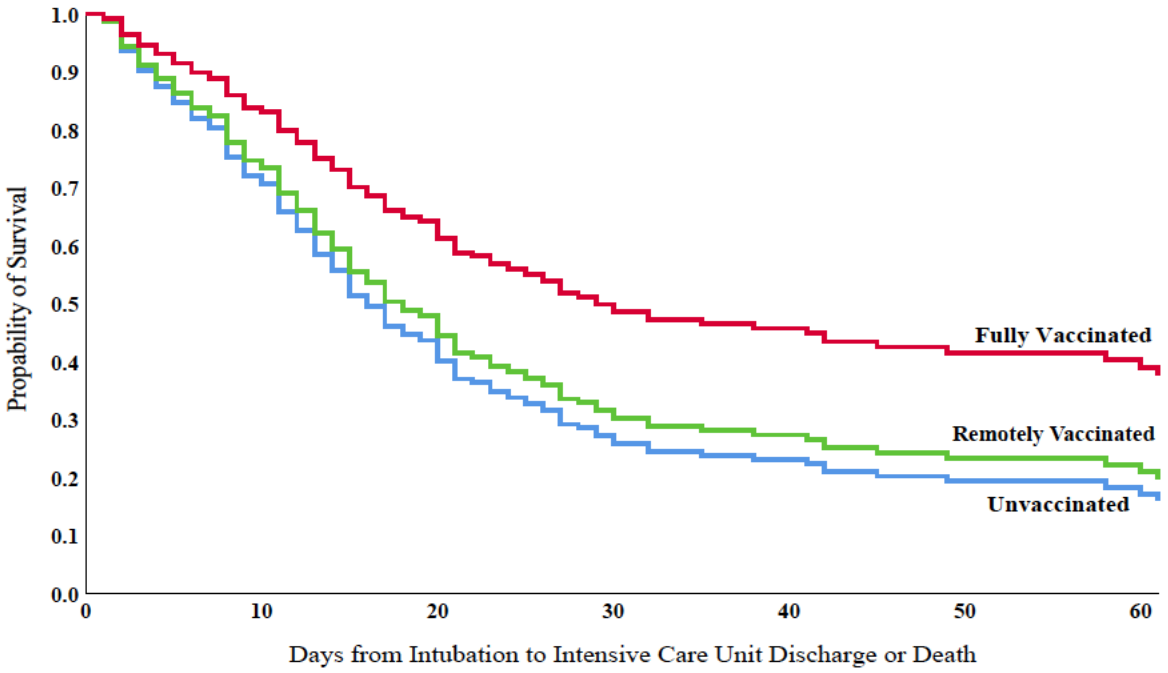

No. at risk							
Fully Vaccinated	26	22	12	8	7	4	4
Remotely Vaccinated	32	22	14	2	1	1	1
Unvaccinated	206	142	77	42	32	20	14

eFigure 1. Survival Curves in the Sensitivity Analysis by Separating the Control Group Into Unvaccinated and Remotely Vaccinated Patients (i.e., Those Vaccinated More Than Five Months Before Intubation). For time-to-event analysis from intubation to all-cause intensive care unit-mortality, we used a Cox proportional-hazards regression model including vaccination status, age, comorbidity and baseline Sequential Organ Failure Assessment score on the day of intubation and we plotted the corresponding Cox-generated estimated survival curves. The hazard ratio for the association between status of fully vaccinated patients, as opposed to unvaccinated, and intensive care unit-mortality was 0.53 (95% confidence intervals 0.31-0.93); p=0.03. One patient was vaccinated less than 14 days before intubation and was excluded from this sensitivity analysis.

© 2022 Grapsa E et al. *JAMA Network Open.*

eTable 5. Cox Proportional Hazards Regression Model to Isolate the Contribution of Vaccination Status, Age, Comorbidity, SOFA Score, and Presence of Severe ARDS on the Day of Intubation (Independent Variables) to All-Cause Intensive Care Unit Mortality (Dependent Variable)

	**Hazard ratio**	**95% confidence intervals**	**p value**
Full vaccination status (versus control)	0.58	0.34-1.00	0.05
Age (increments of 1)	1.02	1.01-1.04	0.002
Comorbidity (any versus none)	1.19	0.80-1.75	0.39
SOFA score (increments of 1)	1.23	1.16-1.32	<0.001
Severe ARDS (versus non-severe ARDS)[a]	1.52	1.11-2.09	0.01

Abbreviations: SOFA, sequential organ failure assessment; ARDS, acute respiratory distress syndrome.

[a]Severe ARDS was defined according to the Berlin definition.[7]

© 2022 Grapsa E et al. *JAMA Network Open.*

eTable 6. Cox Proportional Hazards Regression Model to Isolate the Contribution of Vaccination Status, Age, Comorbidity, and SOFA Score on the Day of Intubation (Independent Variables) to All-Cause Mortality by Day 28 Following Intubation (Dependent Variable)

	**Hazard ratio**	**95% confidence intervals**	**p value**
Full vaccination status (versus control)	0.40	0.21-0.75	0.004
Age (increments of 1)	1.02	1.01-1.03	0.008
Comorbidity (any versus none)	1.58	1.04-2.40	0.03
SOFA score (increments of 1)	1.28	1.20-1.37	<0.001

Abbreviations: SOFA, sequential organ failure assessment.

© 2022 Grapsa E et al. *JAMA Network Open.*

eTable 7. Cox Proportional Hazards Regression Model to Isolate the Contribution of Vaccination Status, Age, Comorbidity, and SOFA Score on the Day of Intubation (Independent Variables) to All-Cause In-Hospital Mortality (Dependent Variable)

	**Hazard ratio**	**95% confidence intervals**	**p value**
Full vaccination status (versus control)	0.63	0.38-1.03	0.07
Age (increments of 1)	1.02	1.00-1.03	0.02
Comorbidity (any versus none)	1.31	0.90-1.90	0.16
SOFA score (increments of 1)	1.21	1.14-1.29	<0.001

Abbreviations: SOFA, sequential organ failure assessment.

© 2022 Grapsa E et al. *JAMA Network Open.*

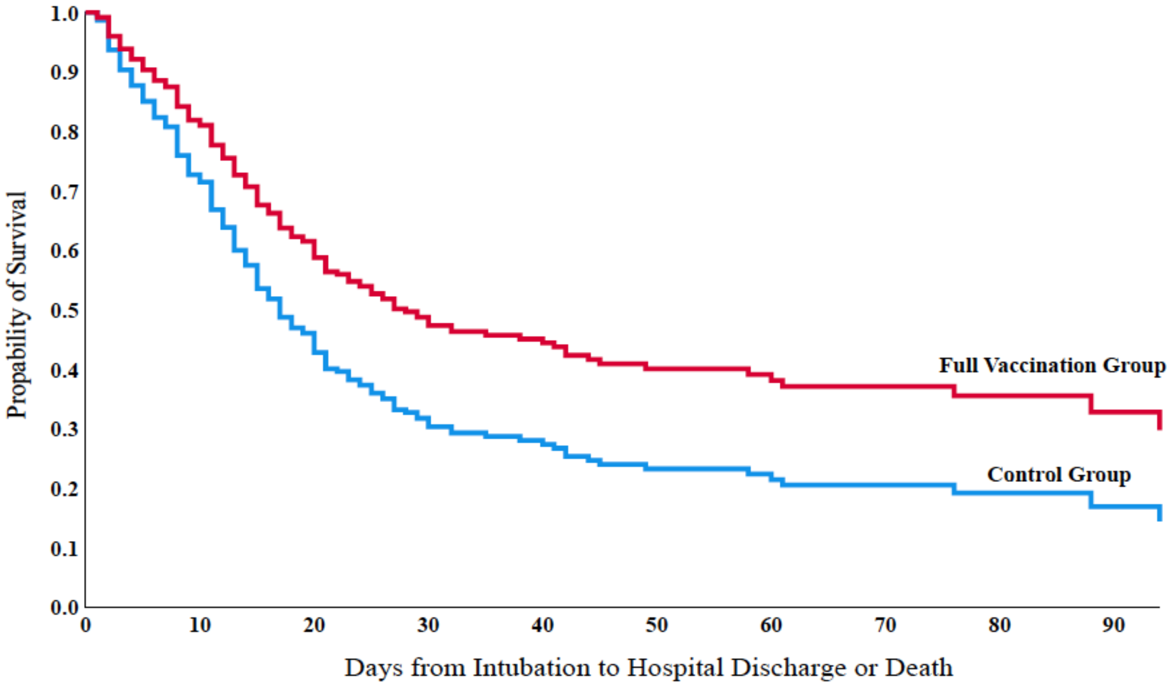

No. at risk										
Full Vaccination Group	26	22	15	12	10	6	6	5	2	1
Control Group	239	168	101	67	42	29	23	19	11	7

eFigure 2. Survival Curves of Patients Included in the Full Vaccination vs Control Group. For time-to-event analysis from intubation to all-cause in-hospital mortality, we used a Cox proportional-hazards regression model including vaccination status (full versus control), age, comorbidity and baseline Sequential Organ Failure Assessment score on the day of intubation and we plotted the corresponding Cox-generated estimated survival curves. The hazard ratio for the association between full vaccination, as opposed to control, and in-hospital mortality was 0.63 (95% confidence intervals 0.38-1.03); p=0.07.

© 2022 Grapsa E et al. *JAMA Network Open.*
